# Repair of exposure and fracture of the porous high-density polyethylene framework after ear reconstruction

**DOI:** 10.1186/s13005-022-00345-y

**Published:** 2022-12-16

**Authors:** Chenyan Jiang, Bin Chen, Lixing Lu, Xiaojun Yan, Bin Yi, Runjie Shi

**Affiliations:** 1 Department of Otolaryngology-Head and Neck Surgery, Shanghai Ninth People’s Hospital, Shanghai Jiao Tong University School of Medicine; Institute of Otology, Shanghai Jiao Tong University School of Medicine, 639 Zhizaoju Road, Shanghai, 200011 China; 2 Shanghai Key Laboratory for Transitional Medicine of Nose and Ear Diseases, Shanghai, 200011 China

**Keywords:** Ear reconstruction, Framework exposure, Framework fracture, Tissue flap

## Abstract

**Objective:**

To assess the repair method of exposure or fracture of the porous high-density polyethylene ear framework after total auricle reconstruction.

**Study design:**

A prospective case study.

**Methods:**

From April 2018 to October 2021, 11 patients with framework exposure or fracture after total auricle reconstruction were admitted to the hospital for repair. In these 11 patients, the repair was performed using (1) a temporal muscle flap combined with free skin graft in 5 patients, (2) a mastoid fascia flap combined with free skin graft in 2 patients, (3) a simple local skin flap in 1 patient, (4) combination of a temporalis muscle flap and a mastoid fascia flap together with free skin graft in 2 patients, and (5) a Su-Por helix material combined with a temporal muscle flap and free skin graft in 1 patient.

**Results:**

After follow-up for 3–36 months, except for one patient in whom local exposure again occurred at the same site, the framework was in a good shape in the other patients, and all the skin graft survived.

**Conclusion:**

The defect of the upper part of the auricle can be repaired using a temporal muscle flap combined with temporal muscle fascia and skin graft. The defect of the middle and lower part of the auricle can be repaired using a mastoid fascia flap combined with skin graft. For framework fracture, the damaged site can be first strengthened with another ear material and then combined with the adjacent fascia flap and free skin graft.

## Introduction

The deformity or absence of ears can cause great psychological distress to patients and severely affect their daily activities and life [1]. Total auricle reconstruction provides an effective treatment method for these patients. At present, autologous costal cartilage and porous high-density polyethylene are mainly used as implant materials in clinics [2, 3]. By using porous high-density polyethylene, total auricle reconstruction can be completed in one stage, thereby reducing the treatment period. It can also avoid the risk of thoracic injury caused by severing of the costal cartilage and prevent instability due to cartilage absorption in long-term use [4]. However, this artificial material is relatively hard, and complications such as skin ulceration and framework exposure can easily occur after operation. Moreover, it is difficult to repair the exposed framework, which may further affect the constructed auricle shape. In severe cases, the framework may have to be removed, which will cause both physical and psychological trauma to the patients and lead to economic loss [5]. Therefore, ear plastic surgeons have been focusing on investigating how to effectively manage such complications, reduce surgical trauma as much as possible, and ensure the integrity of the reconstructed ear.

For this purpose, this study aimed to find the best treatment strategy for patients with ear framework exposure or fracture. The study included 11 patients in whom framework exposure or fracture occurred after one-stage total auricle reconstruction to assess the clinical effect of repairing the defect with a muscle flap, a fascia flap, or a local flap.

## Materials and methods

Eleven patients were recruited in the study from April 2018 to October 2021. These patients included six males and five females, with an age range of 6–53 years; four patients underwent left ear reconstruction, and seven patients underwent right ear reconstruction. Framework exposure occurred in 10 patients, while framework rupture occurred in 1 patient. Framework exposure occurred twice in the same area in one patient. The exposed parts of the framework were the upper part of the helix in four patients, the middle part of the helix in two patients, the end part of the helix in three patients, the concha cavity in three patients, the triangular fossa in one patient, and the antihelix in two patients. The size of the defect area ranged from 0.3 cm × 0.3 cm to 3.0 cm × 3.0 cm. The exposure time after operation ranged from 1 to 28 months. An adjacent muscle flap, a fascia flap combined with free skin graft, and a local flap were used to repair the defect, as shown in Table 1. In accordance with the current bioethical guidelines, a written informed consent form was signed by all patients, and the study was approved by the Institutional Review Board of Shanghai Ninth People’s Hospital (Approval Number: 2016–138-T87).

**Table 1:** Clinical data of the 11 study patients

Age (yr)	Gender	Time of exposure / fracture (months)	Position	Size (cm)	Flap type
12	Female	28	Upper part of helix	0.8 × 0.8	Temporal muscle flap
6	Female	1	Upper part of helix Concha cavity	0.3 × 0.3 3.0 × 3.0	Temporal myofascial flap Mastoid fascia flap
11	Female	25	Upper foot of antihelix	0.7 × 0.8	Temporal muscle flap
9	Male	24	Crus of helix	0.7 × 0.7	Local flap
6	Male	8	Concha cavity	1.2 × 1.2	Temporal muscle flap
53	Female	10	Middle part of helix	0.7 × 0.6	Temporal muscle flap
14	Male	2	Concha cavity Antihelix	1.5 × 1.5	Mastoid fascia flap
11	Male	11	Upper part and crus of helix	1.0 × 1.0 1.0 × 1.0	Temporal myofascial flap Mastoid fascia flap
8	Male	10 6	Upper part of helix Upper part of helix	1.2 × 1.8 1.2 × 1.0	Temporal myofascial flap Temporal muscle flap
7	Male	2	Crus of helix	0.6 × 0.8	Mastoid fascia flap
6	Female	14	Middle part of helix	Fracture	Temporal muscle flap

### Incision design

**Fig. 1:**
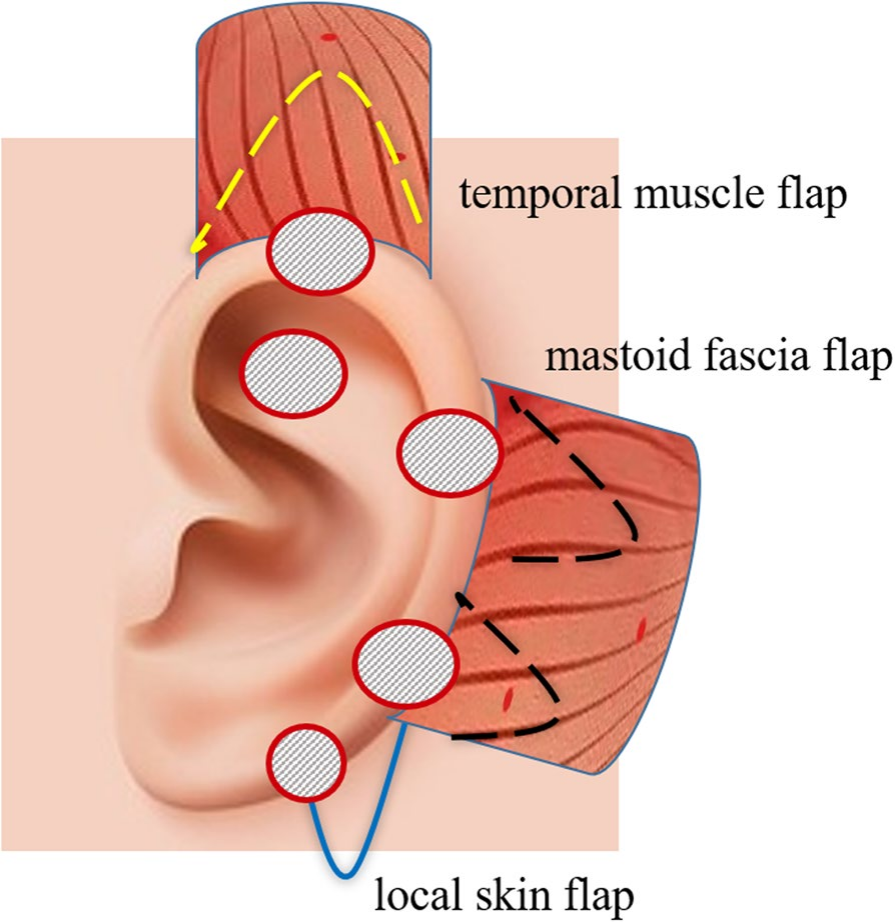
A diagrammatic illustration of the surgical technique. The red circled diagonal stripes represent the exposed parts of the implant. The yellow dashed line represents the prepared temporal muscle flap. The black dashed line represents mastoid fascia flap. The solid blue line represents the prepared local skin flaps

Different incisions were selected according to the exposed or damaged parts of the framework. For the wound near the upper part of the auricle, a longitudinal fusiform incision was made along the edge of the defect and then extended to the previous scalp incision. For the wound near the middle and lower part of the auricle, a transverse fusiform incision was selected and extended to the mastoid area. Extension of the incision was not required for a small-sized defect close to the center of the auricle. The repaired tissue flap was directly turned over to the surface of the framework through the subcutaneous tunnel. All incisions were hardened in the hair as much as possible. The incision length was determined based on the size of the required tissue flap (Fig. 1).

### Debridement

Infected skin and fascia tissue at the wound edge were removed. Scar tissue or granulation tissue, if any, on the surface of the framework was also removed. In case of local infection, the gray-white inflammatory tissue on the surface of the framework was removed, and the superficial part of the material was trimmed until active bleeding was observed on the surface of the framework. Finally, the wound was washed sequentially with iodophor, chloramphenicol, and saline.

### Treatment of different exposure parts

For frame exposure in the upper part of the auricle, the scalp was cut along the previous incision to expose the temporal muscle area. A bottom pedicle temporal muscle flap containing the temporal muscle fascia was designed according to the defect range. Careful attention was given to protect the branches of the temporal artery while preparing the muscle flap. The required tissue flap was designed to be 0.5–1 cm wider than the exposure range of the framework, so as to avoid the secondary material exposure due to fascia contracture later. The temporal muscle flap was turned over and covered the surface of the material without tension. The flap was sutured and fixed with the surrounding previous superficial temporal fascia flap. Free epidermal skin graft was transplanted on the surface (taken from the previous abdominal incision to reduce the recurrence of donor scar), and the skin graft area was packed and pressurized. The dressing was removed after 12–14 days. A mini negative pressure drainage was placed under the scalp, derived from the scalp hair, and removed after 5 days.

For framework exposure in the middle and lower part of the auricle, an incision was made in the mastoid region to prepare the mastoid fascia flap containing the posterior auricular artery and the occipital artery. The upper portion of this area was aligned with the bottom of the previously prepared superficial temporal fascia flap. Careful attention was given to protect the branches of the superficial temporal artery. The fascia flap pedicled at the posterior ear sulcus was turned over and covered the surface of the framework. The epidermal skin graft was transplanted onto the surface of the fascia flap. A mini drainage tube was placed in the ear groove, with its opening near the hairline.

If the framework exposure was located at the end of the helix or the crus of helix, the ductility of the surrounding skin could be used to prepare an axial local flap to directly repair the damaged site.

### Treatment of framework fracture

The most likely region of framework fracture is the highest point of the middle and upper part of the helix. Here, the broken ends of the material were properly trimmed to remove the tissue that may be infected, and a new material was prepared to reinforce the fracture point. The adjacent fascia flap was prepared to cover the surface of the reinforcement material. The mini drainage tube was placed in the helix groove, and the free skin graft was transplanted to cover the wound.

### Typical case 1

**Fig. 2:**
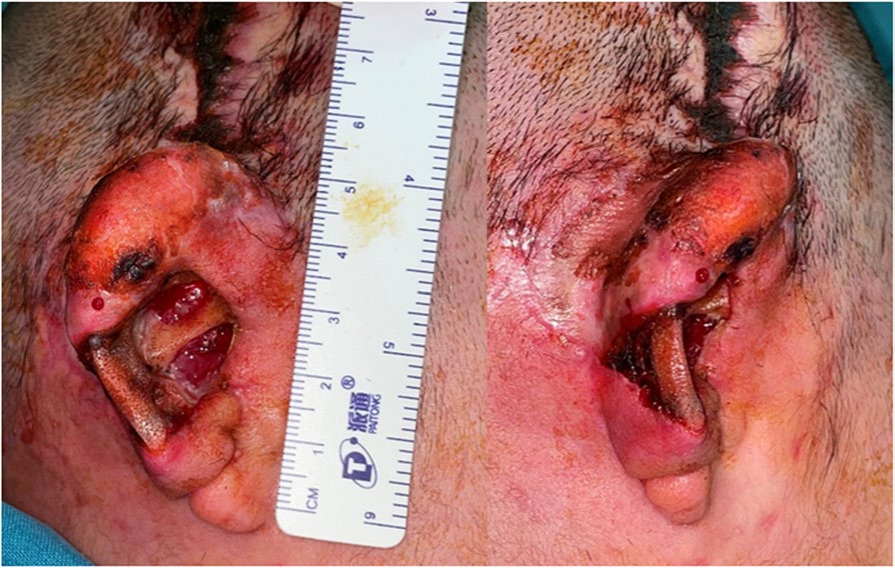
Framework exposed after 3 weeks of one-stage auricle reconstruction with a 3D printed Su-Por. A small skin defect (0.3 cm × 0.3 cm) appeared in the upper part of the helix with the exposure of framework. A large skin defect (3.0 cm × 3.0 cm) was observed in the auricular cavity and middle auricle with exposed framework

**Fig. 3:**
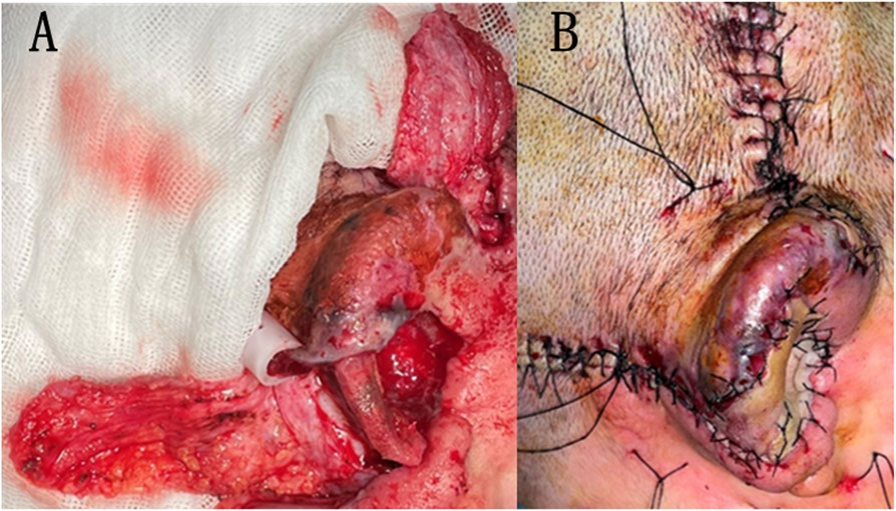
Preparation of the tissue flap and free skin graft. A 2 cm × 3 cm-sized temporal muscle flap and a mastoid fascia flap of approximately 4 cm × 4 cm was made (**A**). The temporal muscle flap was turned over and passed through the skin above the highest point of the auricle to cover the surface of the exposed framework. The mastoid fascia flap was turned forward to the concha, and the epidermal skin graft was transplanted onto their surface. A mini drainage was left under the scalp and auricle, and the cavity was then closed (**B**)

**Fig. 4:**
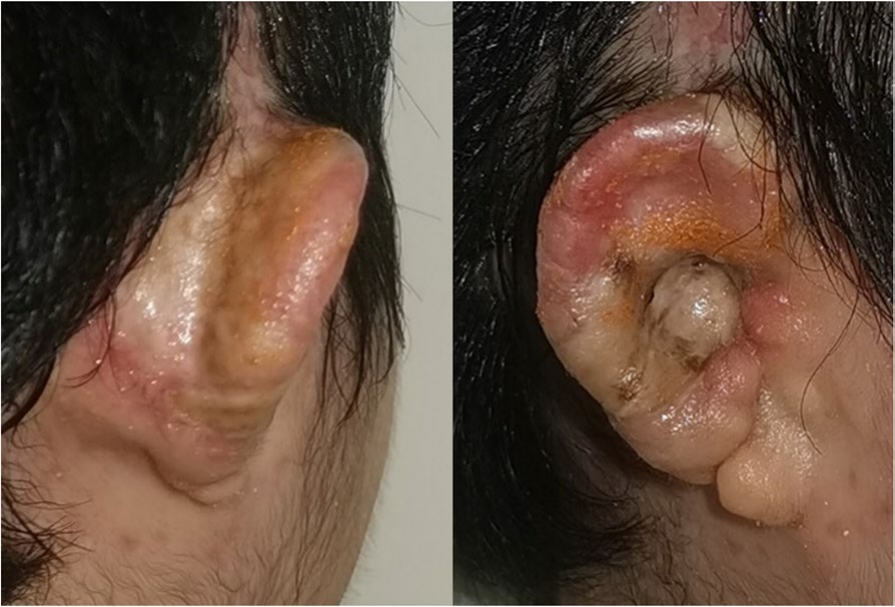
Postoperative images of the repair surgery. The frontal and lateral views at 2 months postoperation shows the fine structure of the reconstructed auricle, and the grafts survived with good color

The patient was a 6-year-old girl who underwent one-stage auricle reconstruction with a 3D printed Su-Por (porous high-density polyethylene) ear framework combined with a temporal fascia flap and simultaneous bone bridge implantation. When the external ear dressing was removed 2 weeks after operation, the auricle was swollen, and blood accumulation with odor was observed in the ear cavum conchae together with a small skin defect of approximately 0.5 cm × 1 cm in size. The temporal fascia was observed in the deepest part of the cavum conchae. Local subcutaneous hematoma in the anterior temporal area with a skin defect of approximately 1 cm × 1 cm in size was also found. Debridement was performed immediately under general anesthesia. During the operation, 4–6 scalp sutures were removed, and a small amount of blood accumulation and blood clots were found under the scalp. The wound was cleaned, and a drainage tube was then placed under the scalp. Suture of the temporoparietal fascia flap was partially removed, and the accumulated subcutaneous blood was squeezed out in the cavum conchae. The temporoparietal fascia flap was used to cover the framework and sutured again. The operation area was sequentially washed with iodophor, hydrogen peroxide, and saline. Bleeding was completely stopped with bipolar electrocoagulation, and a mini drainage tube was placed in the helix groove. Finally, a gelatin sponge was used to cover the exposed fascia surface of the cavum conchae, and the total auricle was wrapped with an iodoform gauze. After the operation, the defect area was disinfected with benzalkonium chloride and coated with epidermal growth factor every day. One week later, we found that the defect did not improve, and the scope of the defect skin expanded to approximately 3 cm × 3 cm in size; moreover, to make the situation worse, the framework under the concha was exposed. A repair operation was performed under general anesthesia. During the operation, multiple scabs were found around the upper one-third of the auricle and the temporal area. After the scabs were cleaned, a small skin defect (0.3 cm × 0.3 cm) appeared in the upper part of the helix with framework exposed. The scalp incision was cut along the previous incision to expose the temporal muscle. A 2 cm × 3 cm-sized temporal muscle flap pedicled below was made. We turned over the prepared temporal muscle flap and passed it through the skin above the highest point of the auricle to cover the surface of the exposed framework. The temporal muscle flap was sutured and fixed with the surrounding fascia flap, and an epidermal skin graft was transplanted onto its surface. We excised approximately 3 mm skin around the epidermal defect of the cavum conchae, removed the deep necrotic granulation and fascia tissue, and appropriately thinned the exposed framework. A transverse incision of approximately 6 cm was made in the mastoid area along the skin defect to expose the occipital muscle, and a mastoid fascia flap of approximately 4 cm × 4 cm was made and turned forward to the concha. The mastoid fascia flap was sutured and fixed on the surface of the exposed framework, and an epidermal skin graft was transplanted above it. One mini drainage was left under the scalp and auricle. Proper packaging and fixing of the wound area were performed with a Vaseline gauze and 1% chloramphenicol liquid gauze dressing (Figs. 2, 3 and 4).

### Typical case 2

**Fig. 5:**
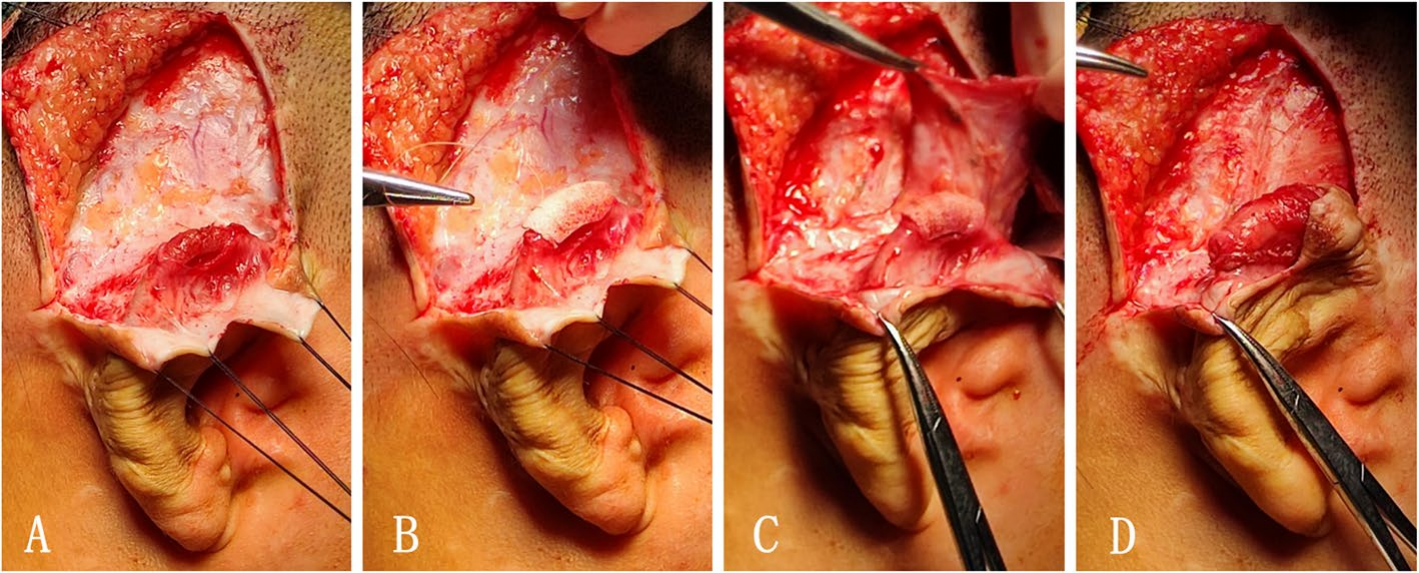
Treatment of framework fracture. A fracture at the highest point of the helix was detected (**A**). Another Su-Por helix framework was cut to approximately 1.5 cm in length to reinforce and fix on the surface of the fracture site (**B**). A pedicled mastoid fascial flap with a size of 3 cm × 4 cm was made (**C**). The mastoid fascial flap was turned over to wrap the reinforced material and sutured with the peripheral superficial temporal fascia (**D**)

The patient was an 8-year-old girl who underwent one-stage auricle reconstruction with a Medpor framework combined with a temporoparietal fascia flap. Twenty months after operation, her parents found that the location of the reconstructed auricle leaned back, and there was a sense of ladder at the highest point of the helix, but the surface skin was intact. We severed approximately 4 cm length along the previous scalp incision and continued the incision beneath the helix sulcus. When the subcutaneous tissue was separated and loosened, a crack in the fascia and a fracture at the bottom of the framework was found. The fascia wrapping the broken framework was cut, and the broken ends of the framework were trimmed and sutured to fix them. We took another Su-Por helix framework and cut it approximately 1.5 cm in length to make it similar to the fractured part. The edge of the material was made smoothly. It was then used to reinforce and fix on the surface of the fracture site. We prepared a pedicled mastoid fascial flap with a size of 3 cm × 4 cm and turned it over to wrap the reinforced material. The mastoid fascial flap was sutured with the peripheral superficial temporal fascia, and a mini drainage tube was left in the helix groove. Finally, an epidermal skin graft of 5 cm × 3 cm in size was transplanted on the surface of the fascia. Proper packaging and fixing were then performed (Fig. 5).

## Results

The 11 patients were followed up for a range of 3 months to 3 years. All the grafts survived with good color, and the auricle showed a fine structure. No other complications occurred. The patients and their relatives were satisfied with the outcome of repair surgery. In one case, because of wearing spectacles for a long time, local framework exposure occurred again at the highest point of helix 6 months after the operation, and surgical repair was performed again. In the last 6 months of the follow-up, the patients have shown good recovery. None of the other patients had framework re-exposure.

## Discussion

Framework exposure is the greatest risk and challenge faced by surgeons who choose to use ear framework materials to complete total auricle reconstruction. The incidence of alloplastic material exposure in ear reconstruction ranged from 7.3 to 13.24%, but in almost all cases, exposure was actually caused by an unstable tissue covering the implant and not the implant itself. Careful attention on some technical issues during the operation and special nursing care after the operation may be helpful to prevent framework exposure. The common causes of framework exposure are as follows: ① The soft tissue that wraps the framework is too small. The size of the superficial temporal fascia and the transplanted skin graft above the framework is too small or the tension between them is too large. When this occurs, poor local blood supply and ischemic necrosis of the fascia and skin may occur in the early stage. This will also lead to the exposure of the material at the highest point of the helix and other convex parts due to fascia and skin contraction in the late stage. Therefore, while preparing fascia flap and skin graft, the size should be large enough to make the contact between superficial temporal fascia and framework tension-free. The preparation of the fascia flap should not only include the superficial temporal fascia but also include the deep temporal fascia. If necessary, it can include the galea aponeurotica to ensure sufficient blood supply to the fascia flap [6]. Regardless of use of the fascial flap or free skin graft, there should be no tension when covering the framework, especially at the earlobe junction. The thickness of the skin differs between the two parts, which makes it easy to cause skin contracture and cracking. ② Collision and extrusion by force. The skin covered on the surface of the ear framework is transplanted from the abdomen or thigh root. It has poor collision resistance and repair ability. Collision, scratching, and extrusion will lead to skin damage and exposure or fracture of the framework. The exposure of the framework after 6 months of the operation was mostly related to external force collision or long-term compression. We suggest that patients sleep with soft pillows or earmuffs and avoid collision sports in daily life. We found that the most common site for framework exposure is the helix. Therefore, for children with a thin fascia flap, a long strip of skull periosteum can be prepared to wrap around the helix framework for strengthening and protection. ③ Postoperative infection and hematoma. Early framework exposure is usually related to infection and hematoma, and the wound usually expands rapidly. In this group of patients, the size of framework exposure caused by infection increased from 0.5 cm × 1.0 cm to 3 cm × 3 cm only in 1 week. During the first 3 months after operation, the incision and skin graft area were still in the healing stage. There may be focal necrosis or infection in the skin flap and fascia flap, which needs close observation. Once hematoma is detected, hematoma and blood clot should be removed promptly, and drainage should be placed to avoid secondary infection. For the wider and thicker blood scab, necrosis and infection under the scab may occur, and a skin defect will be formed after removal, resulting in framework exposure. The scab should be excised in the early stage, and the wound should be treated with some anti-inflammatory and epidermal growth factor gel, such as benzalkonium chloride disinfection or gentamicin flushing. ④ Improper stitching and packing. If the alignment of the incision suture is poor or too sparse, the epidermis can easily crack after removing the suture. While packing the wound, an excessive pressure is induced by the dressing and bandage, which can affect the blood supply to the fascia flap and skin flap, resulting in local necrosis of the skin flap or the fascia flap and framework exposure. Therefore, when dressing the wound, the triangular fossa, boat fossa, and concha cavity should be filled with small strips of gauze to ensure the appearance of the fine structure of the auricle, and the helix should be wrapped with two layers of Vaseline gauze for protection. The total reconstructed auricle should be packed with an antibiotic gauze with proper pressure. Generally, the packing should be removed after 12–14 days. ⑤ The end edges of the framework are too sharp. The flexibility of the ear material is worse than that of autologous cartilage, and all edges should be trimmed smoothly, especially the ends of the framework and the crus of the helix. If these two parts are exposed, they are mostly related to the sharp end of the material. A small burr on the surface of the material can cause rupture of the tissue flap and lead to framework exposure. For treating earlobe junction, the residual earlobe should be separated to form a capsule, and the end of the framework should be fixed in the residual earlobe soft tissue as much as possible. ⑥ The ear framework is not stably fixed. During the operation, the ear framework needs to be accurately fixed on the periosteum of the lateral cranial wall to form an ideal cranioauricular angle. Stable framework placement is crucial to bear the postoperative flap contraction and maintain a good shape. It can also avoid the fracture of the framework due to the movement or loosening of implants by force in the later stage. To strengthen the stability of the implants, a sharp knife can be used to punch holes at the upper, middle, and lower parts of the ear base implants, and the framework can be sutured through these holes and fixed stably on the side wall of the skull. At present, 2-piece porous implants (Medpor/Su-Por) are prepared from a thin helical rim and thick ear base that are melted together to create the final form of the implant. An additional material is soldered between the two pieces to strengthen the implant; however, when a joint is made between two pieces, there is a weak point that can become crack due to stress over time [7]. In 2015, an American surgeon Dr. Lewin created a 3D single intricately shaped piece from porous polyethylene; the base of the 3D ear implant was flat, which can fit well on the skull surface and thus greatly improve the stability of the framework. This implant was reported to reduce the risk of implant fracture to 3%.

Choosing the appropriate tissue flap to repair the damaged framework is very important for the recovery of postoperative appearance and to avoid the occurrence of other complications. At present, the common tissue flaps that can be used for repairing ear framework exposure or fracture include the deep temporal fascia flap, temporal muscle flap, mastoid fascia flap, and local skin flap [8–10].

In the present patient group, the superficial temporal fascia flap was used, and the deep temporal fascia was removed in some patients. This makes it more difficult for us to choose the appropriate repair methods. The temporal muscle flap is a pedicled island flap. The tissue flap contains branches of the superficial and deep temporal arteries and veins, with good blood supply and high survival rate [11]. Clinically, it is commonly used to repair soft tissue defects in the orbital region, skull base, and temporomandibular joint [12–14]. The temporal muscle is adjacent to the auricle and has sufficient tissue to apply; thus, it can be used as an ideal method to repair the exposure of auricle framework [15]. In the present study, the defect of the upper part of the helix was mostly repaired with a tissue flap containing the deep temporal fascia and a part of temporal muscle, which could reduce the risk of secondary exposure due to fascia flap contracture. In this group, the deep temporal fascia flap was chosen for repair by one child at the first time, and the highest point of the helix was exposed again 6 months later due to wearing spectacles for a long time. The temporal muscle flap was used to repair at the second time. The child has been followed up for 6 months, and the shape of the auricle has recovered well. When preparing the temporal muscle flap, the incision can be directly made upward from the exposed and damaged helix skin surface to the temporal area or directly cut from the previous scalp incision to expose the temporal muscle. One should be careful to separate the fascia flap to avoid damaging the superficial temporal artery and vein branches. When the fascia flap needs to be folded back, careful attention should be given to protect the pedicle by avoiding pressure, especially while moving through the skin tunnel to repair the wound.

For patients with framework exposure in the middle and lower part of the auricle, the mastoid fascia flap can be selected for repair. In 1991, by autopsying the mastoid region, Park found that the mastoid fascia was divided into the superficial and deep layers. The superficial layer, that is, the continuation of the galea aponeurotica, was supplied by the posterior auricular artery, the posterior branch of the superficial temporal artery, and the occipital artery. The deep layer is also called the innominate fascia [16]. Subsequently, Park used the mastoid fascial flap to complete the reconstruction of microtia with framework for the first time. The anterior half of the auricle was wrapped with a skin flap, and the posterior half was covered with a mastoid fascial flap and free skin graft, which reduced the incidence of framework exposure [17]. The maximum preparation size of the mastoid fascia flap can reach 8 × 6 cm, and the mastoid region has a rich vascular network, which makes it easy for the flap to survive. Thus, it can adequately repair a large area of framework exposure, especially in the lower two-thirds of the auricle [18, 19]. When preparing the mastoid fascia flap, the incision can extend from the defect area parallel to the mastoid region, the lower part can be separated close to the surface of sternocleidomastoid muscle, and the upper part can be separated close to the surface of skull.

The application of a local flap is limited due to the lack of definite blood supply and limited supply area. In this patient group, only one defect near the lower part of the helix was repaired by an axial local flap in the mastoid region. There are only a few reports in the literature, such as the use of a preauricular flap to repair the exposure of cartilage framework at the triangular fossa by Jeremie [20].

## Conclusion

After ear reconstruction with artificial materials, if framework exposure or damage occurs, it should be repaired promptly. During the repair operation, the wound area must be thoroughly debrided, and infected tissues, granulation, scar tissues, and infected artificial materials should be removed. For the damaged framework, another material can be used to reinforce the breakage point. According to the defect location and size, the temporal muscle flap, mastoid fascia flap, and local flap can be selected to repair the wound. When preparing the tissue flap, the blood vessels should be protected, and the range of the flap should be large enough to avoid secondary exposure due to retraction in the later stage. Drainage should be placed in the donor area to avoid hematoma. An epidermal skin graft should also be prepared and sutured at the defect site without tension.

## Data Availability

Not applicable.
